# Morphologic spectrum of the epithelial tumors of the male and female urethra

**DOI:** 10.1007/s00428-023-03565-y

**Published:** 2023-05-26

**Authors:** Alessia Cimadamore, Antonio Lopez-Beltran, Liang Cheng, Rodolfo Montironi

**Affiliations:** 1 Institute of Pathological Anatomy, Department of Medicine, University of Udine, Udine, Italy; 2 Department of Surgery, Cordoba University Medical School, Cordoba, Spain; 3 Department of Pathology and Laboratory Medicine, Brown University Warren Alpert Medical School, Lifespan Academic Medical Center, and the Legorreta Cancer Center at Brown University, Providence, RI USA; 4 Molecular Medicine and Cell Therapy Foundation, c/o Polytechnic University of the Marche Region, Via Tronto 10, 60126 Ancona, Italy

**Keywords:** Urethra, Urothelial carcinoma, Squamous cell carcinoma, Conventional adenocarcinoma, Clear cell adenocarcinoma, Accessory glans carcinoma

## Abstract

The classification of the epithelial tumors of the male and female urethra includes benign and malignant neoplasms. Primary urethral carcinomas and adenocarcinomas of the accessory glands are the most relevant tumors, both from the morphologic and clinical point of view. An accurate diagnosis, grading and staging are essential for determining adequate treatment strategies and outcome. Information on anatomy and histology of the urethra is of fundamental importance in understanding the morphology of the tumors, including the clinical importance of their location and origin.

## Introduction

The classification of the epithelial tumors of the male and female urethra includes benign tumors (including tumor-like lesions) and malignant tumors (primary carcinomas and tumors of the accessory gland) (Table 1).

**Table 1:** Epithelial tumors of the male and female urethra

Benign epithelial tumors (including tumor-like lesions)	
Malignant epithelial tumors	
Primary carcinomas	
Tumors of the accessory glands	

The aim of this paper is to describe the morphologic spectrum of the epithelial tumors of the male and female urethra based on a personal series of cases.

## Anatomy and histology of the urethra

**Fig. 1:**
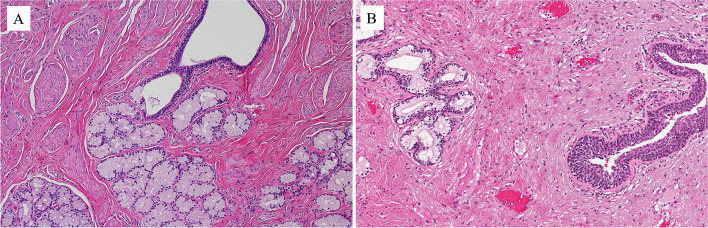
Cowper gland (**A**) and Littré gland (**B**)

The male urethra can be subdivided into three segments: the prostatic urethra lined by urothelium, the membranous urethra located at the level of the urogenital diaphragm, spanning from the prostatic apex to the bulbous urethra, and the penile urethra. The membranous and penile urethras are lined by columnar epithelium. There are two types of accessory glands, the Cowper grand and the Littrè gland (Fig. 1A and B). The first is at the level of the membranous urethra with the duct opening in the bulbous urethra. The Littrè gland is at the level of the penile urethra. Both types of glands are composed of mucosecreting cells. The epithelium of the duct in the Cowper gland is somewhat similar to that in the salivary gland duct.

**Fig. 2:**
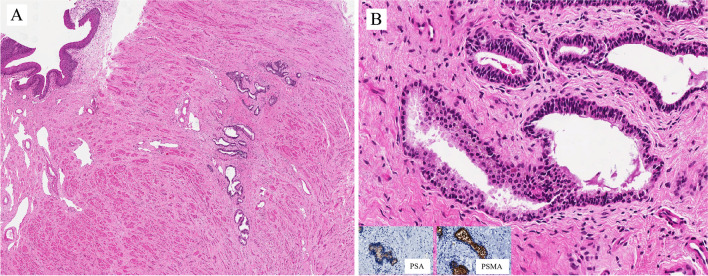
Skene gland

The female urethra is composed of one segment only, spanning from the bladder neck or internal sphincter to the urogenital diaphragm or external sphincter. It corresponds to the location of the prostate gland in men. It is lined by non-keratinizing squamous epithelium. There is one type of accessory gland, called the Skene gland (Fig. 2A). It is lined by a bistratified epithelium similar to that of the normal prostatic ducts and acini, with basal cells and luminal cells (Fig. 2B), the latter positive immunohistochemically for PSA, PSMA (Fig. 2B, inserts), and other prostatic markers.

## Benign epithelial tumors (including tumor-like lesions)

**Fig. 3:**
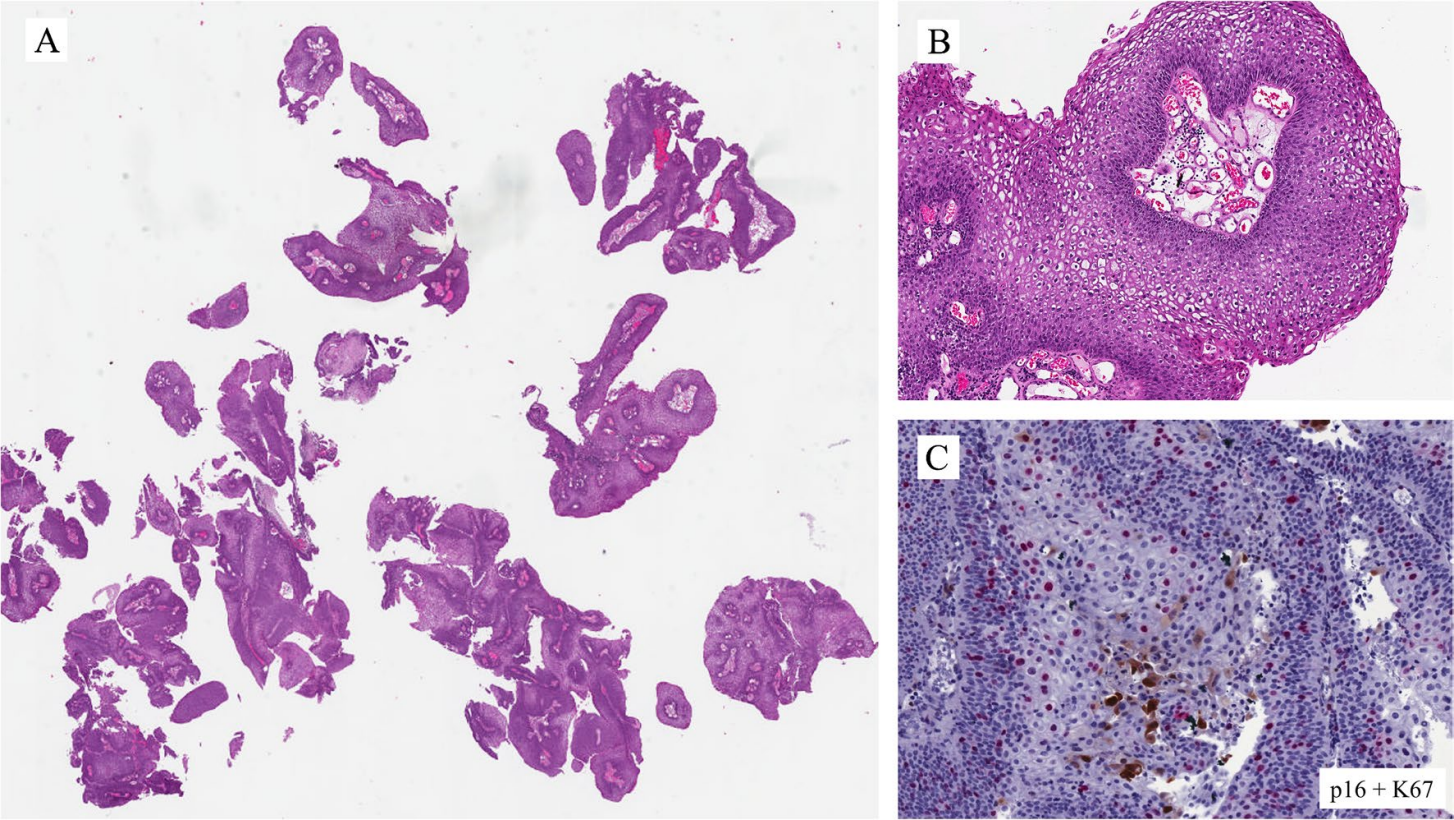
Condyloma acuminatum

**Fig. 4:**
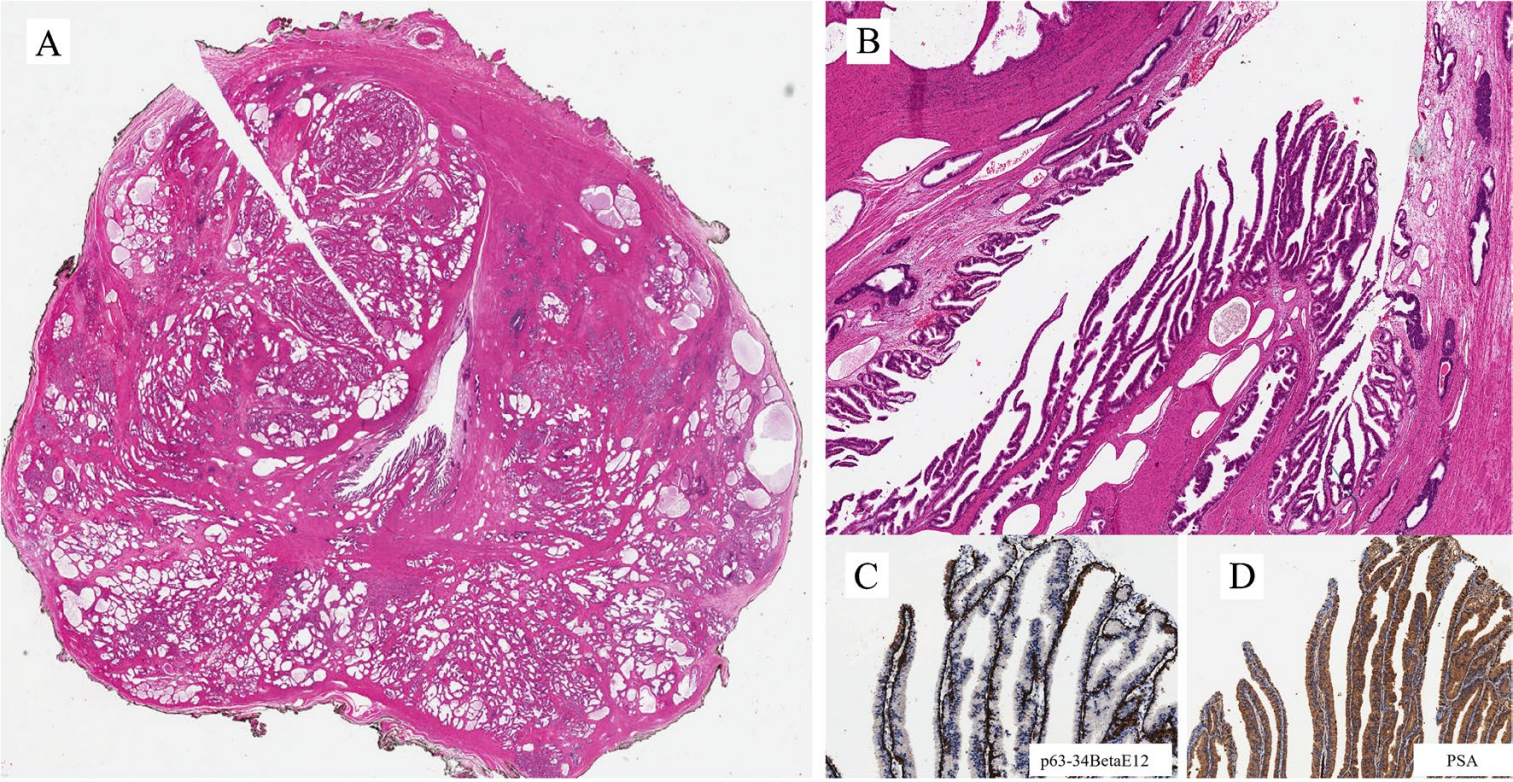
Prostatic-type polyp

Benign epithelial tumors of the urethra are rare in both genders. Tumors and tumor-like lesions occurring in males are similar to those in the female urethra: villous adenoma, urothelial papilloma, squamous papilloma, nephrogenic adenoma (NA), and condyloma acuminatum. The prostatic-type polyp occurs in the prostatic urethra. The pathological importance is linked to the fact that benign tumors can enter the differential diagnosis of primary and secondary urethral carcinomas and vice versa. Figures 3 and 4 are examples of benign lesions of the urethra.

Figure 3A shows a typical example of condyloma acuminatum with a papillomatous architecture lined by nonkeratining squamous epithelium with koilocytic cell change (Fig. 3B) and p16 positive immunohistochemically (Fig. 3C). Low-risk human papillomavirus (HPV), i.e., HPV6 was detected in this case.

Figure 4A is an example of the prostatic-type polyp. It is of a benign lesion, seen in the prostatic urethra in a radical prostatectomy specimen, from a patient with prostate cancer. The lesion shows a villous configuration that protrudes into the urethral lumen and a bistratified epithelial lining similar to that present in the prostatic ducts and acini (Fig. 4B): the basal cells positive immunohistochemically for p63 and 34betaE12 (Fig. 4C) and the luminal cells positive for PSA (Fig. 4D). It has to be considered in the differential diagnosis of lesions with a villous configuration, either benign or malignant, of the urethra.

## Primary urethral carcinoma

Primary urethral carcinoma (PUC) is rare: < 1% of all genitourinary malignancies. The annual age-adjusted incidence in the USA is between 1.5 and 4.2 per million. The incidence peaks in the 75–84-year age group. Men are almost three times more likely to develop PUC than women. Patients of African ethnicity are twice as likely to develop PUC than Caucasians [1, 2].

The classification of the PUCs includes urothelial carcinoma (UC), squamous cell carcinoma (SSC), and adenocarcinoma (ADC). UC is the most common primary type (43%), followed by SCC (31%) and ADC (26%) [3, 4]. There are additional extremely rare primary malignant tumors, such as adenosquamous carcinoma, neuroendocrine carcinoma, and undifferentiated carcinoma.

There are differences in terms of sex-specific and anatomic distribution between men and women. Such differences have a clinical significance. In men, the most common histology is UC (77.6%), followed by SCC (11.9%), ADC (5%) and other histologies (5.5%) [5). Carcinomas in the prostatic urethra are primarily UC, while carcinomas in the penile and bulbomembranous urethra are more likely to be SCC (75%). In women, the most common histology is UC (30–45%), followed by ADC (29%) and SCC (19–28%) [5]. Distal urethral and meatus tumors are most commonly SCC (70%), and tumors of the proximal urethra are UC (20%) or ADC (10%).

### Secondary urethral carcinoma

Primary urethral carcinoma should be distinguished from secondary urethral carcinoma (or secondary involvement). Primary and secondary urethral carcinomas can be morphologically similar, their distinction being based on clinical information. The latter usually originates in tumors of the urinary bladder or prostate. Direct extension from vulvar Paget disease and from primary penile (glans) and skin (scrotum or perineum) carcinoma has been observed [6]. Rare cases of metastasis mimicking PUC have been reported [7].

### Urothelial carcinoma

**Fig. 5:**
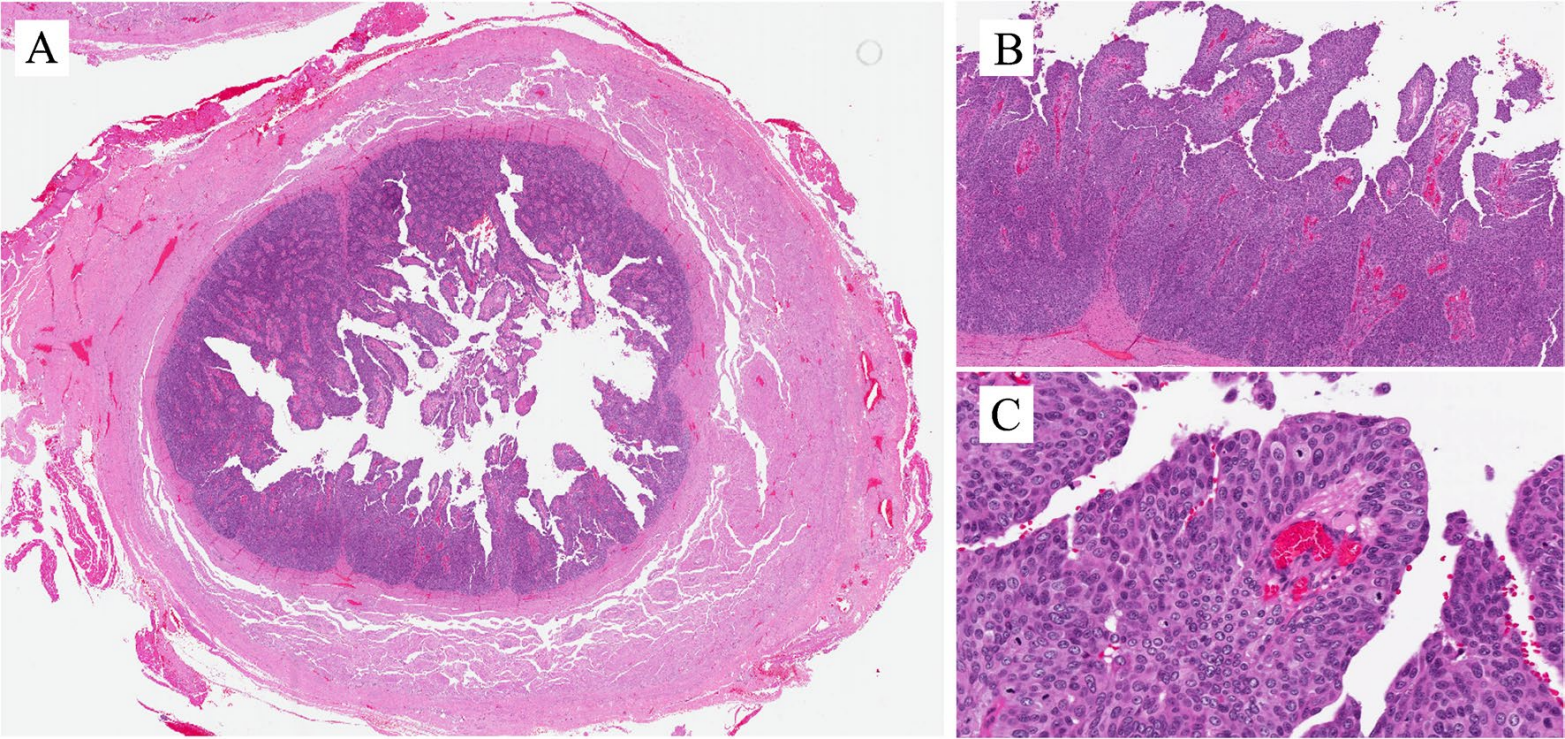
Metachronous urothelial carcinoma in the penile urethra

**Fig. 6:**
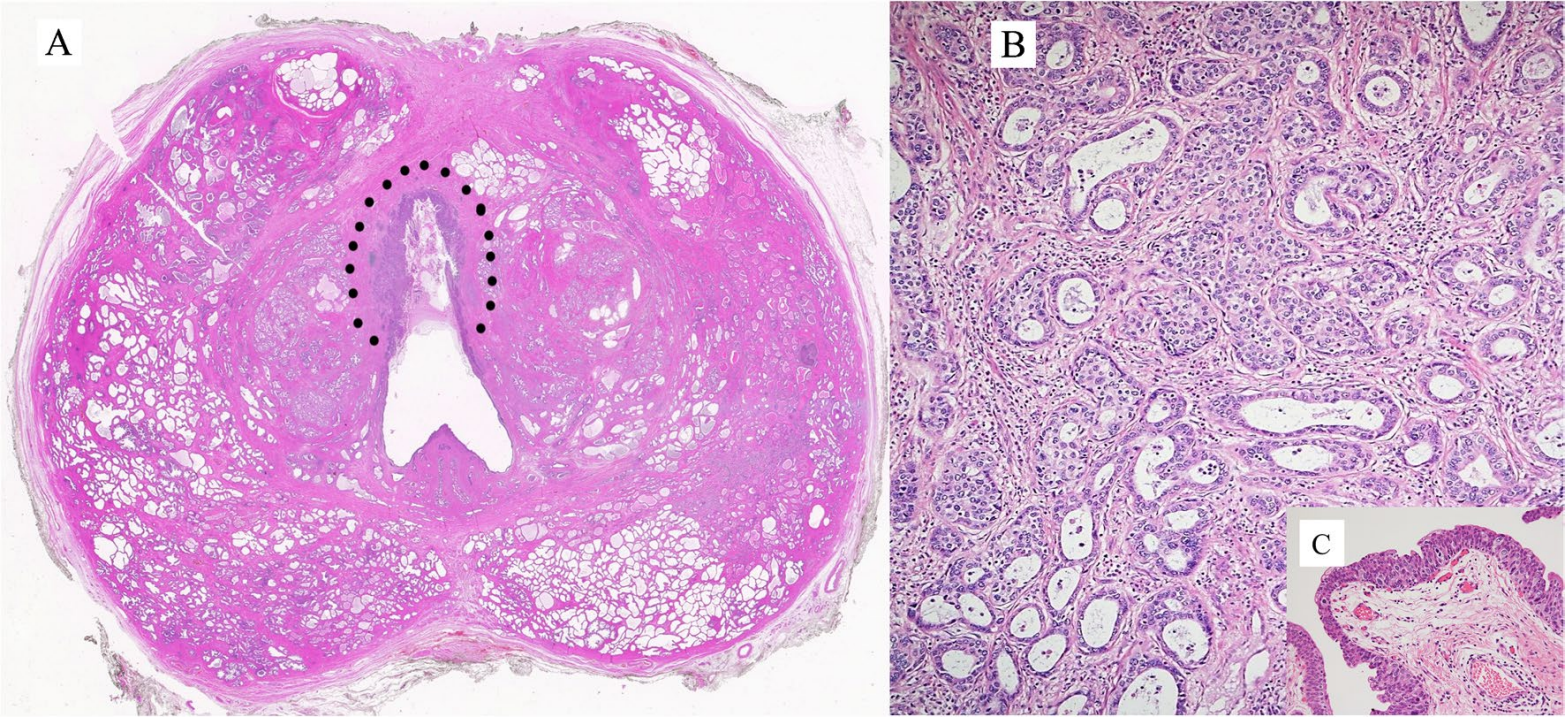
Synchronous urothelial carcinoma of the prostatic urethra

**Fig. 7:**
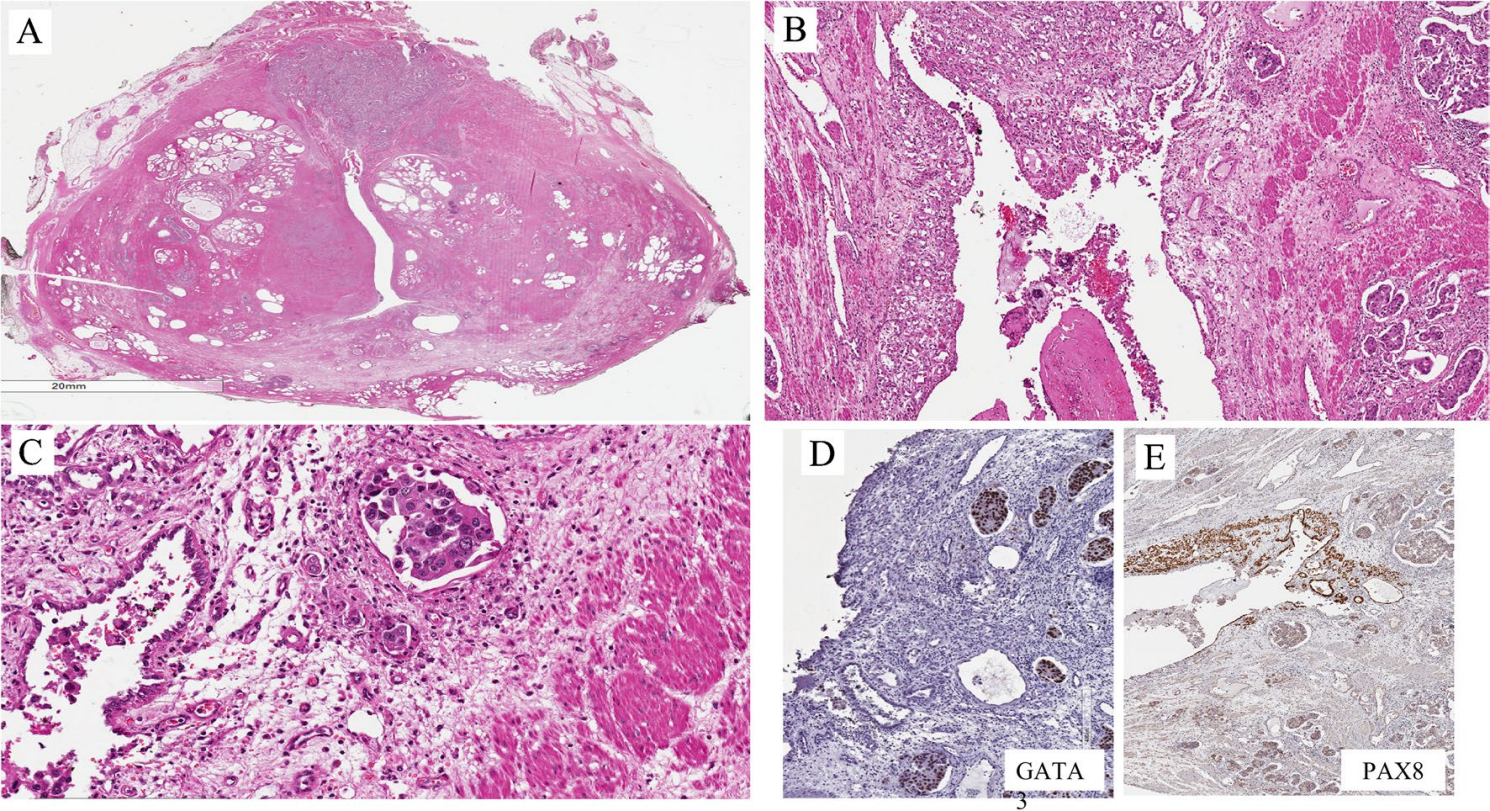
Synchronous urothelial carcinoma of the prostatic urethra and nephrogenic adenoma

Primary UC is rare, compared to secondary involvement by bladder UC (~10–20%). When secondary, it can be synchronous or metachronous to bladder cancer. Primary and secondary urothelial carcinomas of the urethra are morphologically similar. Figures 5–7 are examples of UC involving the urethra.

The images in Fig. 5 are from a penile total urethrectomy specimen in a patient with a previous cystoprostatectomy for pT3a high-grade (HG) UC of the bladder and an initial history of HG papillary carcinoma. It is an example of metachronous UC in the penile urethra (Fig. 5A). Morphologically, it is a papillary UC with fused papillae. The boundary with subepithelial connective tissue is sharp, indicating a non-invasive papillary neoplasm (Fig. 5B). The cells are clearly atypical in a background of altered architecture of the urothelium (Fig. 5C). This allows us to make a final diagnosis and grading. The classification, grading, and variants of UC are identical to those described in the bladder and other parts of the urinary system.

Figure 6 is an example of UC of the prostatic urethra in a cystoprostatectomy specimen in a patient with a long history of recurrent bladder cancer and with incidental prostate cancer. At low magnification, the mucosa of the anterior part of the urethra is ulcerated (Fig. 6A) and shows the presence of solid carcinoma with some microcystic features (Fig. 6B). The tumor is positive for markers typically expressed in UC. It infiltrates the stroma of the prostatic parenchyma. The urothelium adjacent to the invasive UC shows the presence of scattered atypical urothelial cells (Fig. 6C). This represents a form of CIS. The tumor is synchronous to bladder cancer.

Figure 7 shows an additional example of UC of the prostatic urethra in a cystoprostatectomy specimen in a patient with a transurethral resection (TUR)-based diagnosis of high-grade UC invading the muscularis propria of the bladder. At low magnification, the mucosa of the anterior part of the urethra shows the presence of a neoplasm widely invading the anterior part of the prostate (Fig. 7A). It is a high-grade UC with some stromal retraction. Adjacent to this carcinoma, there is a lesion with tubular and microcystic appearance (Fig. 7B). There seems to be some kind of transition from the solid UC and the lesion with tubular and microcystic appearance (Fig. 7C). The question is whether it is a UC with divergent differentiation or a combination of UC and NA. The results of immunohistochemistry show that the neoplasia is GATA3 positive (Fig. 7D) as in UC and the other part is PAX8 (Fig. 7D) as in NA. The message from the case is that primary urethral carcinomas may mimic benign lesions and vice versa.

### Squamous cells carcinoma

Squamous cells carcinoma is similar in histology to SCC at other sites. It is graded on a 3-tiered system and can range in morphology from well differentiated (including the rare verrucous variant) to moderately differentiated (the most common) to poorly differentiated, including basaloid and sarcomatoid. The tumors arising in the distal one-third of the female urethra are frequently low-grade SCC or verrucous carcinoma.

The urethra is occasionally the site of a secondary involvement of a SCC originating from adjacent organs, in a manner similar to that seen with UC: synchronous or metachronous.

**Fig. 8:**
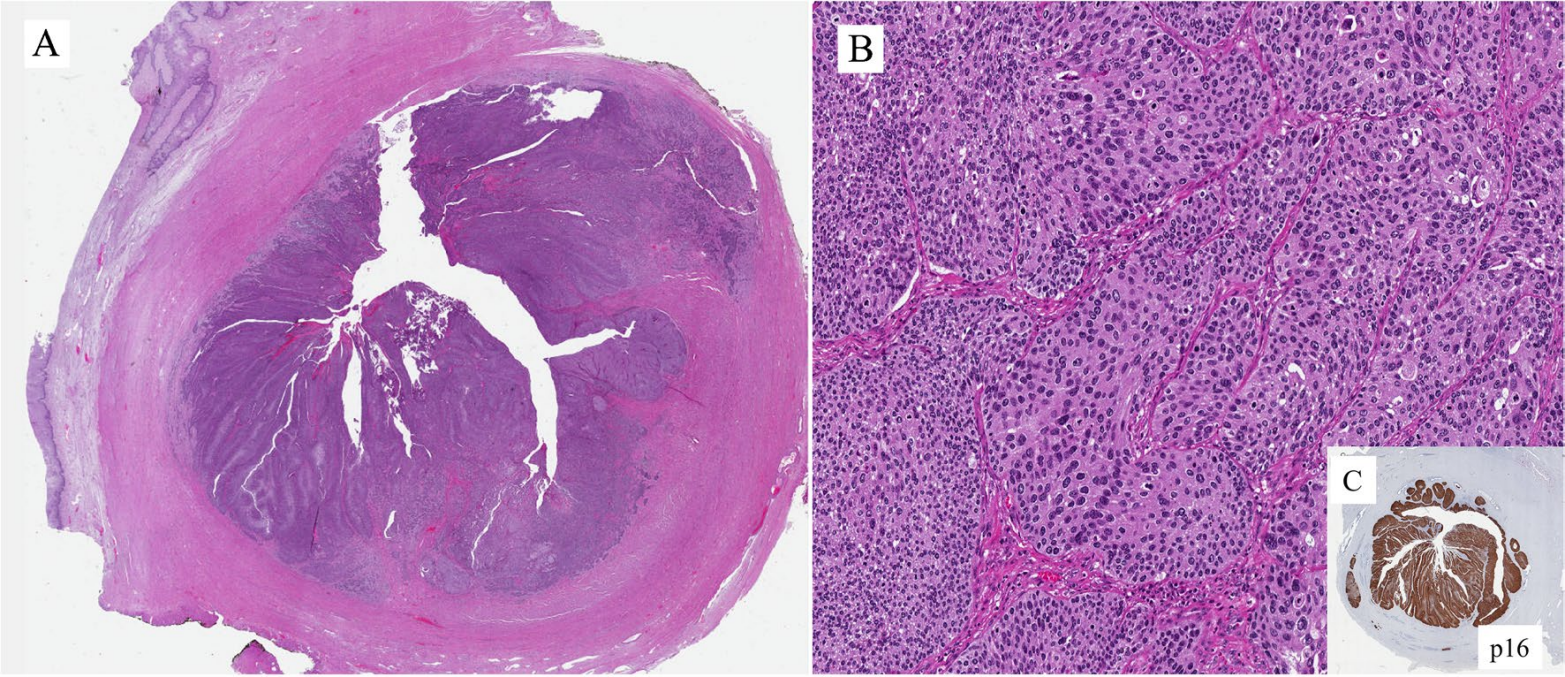
Squamous cells carcinoma in the female urethra

**Fig. 9:**
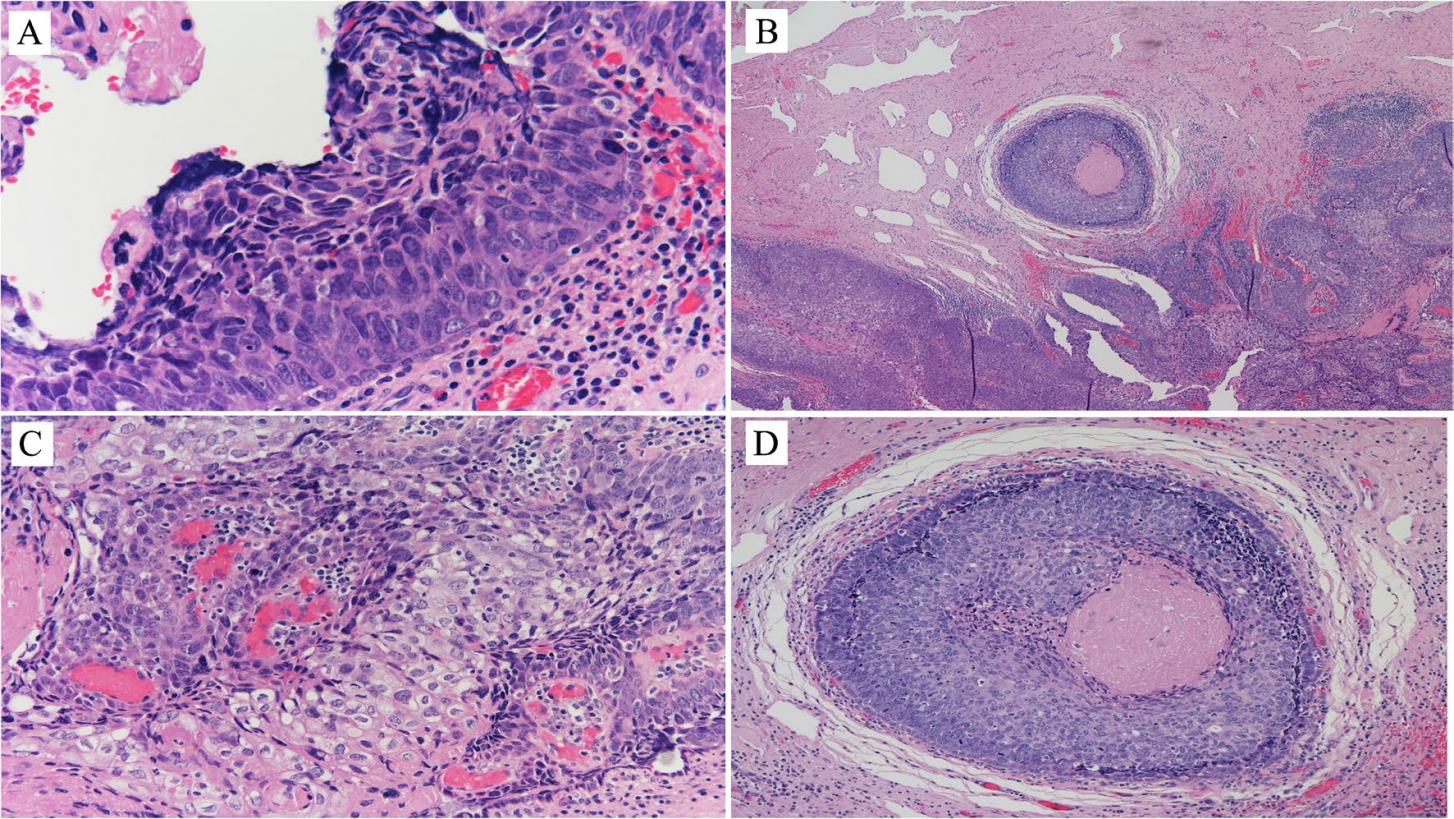
Male urethra: basaloid variant of squamous cells carcinoma

**Fig. 10:**
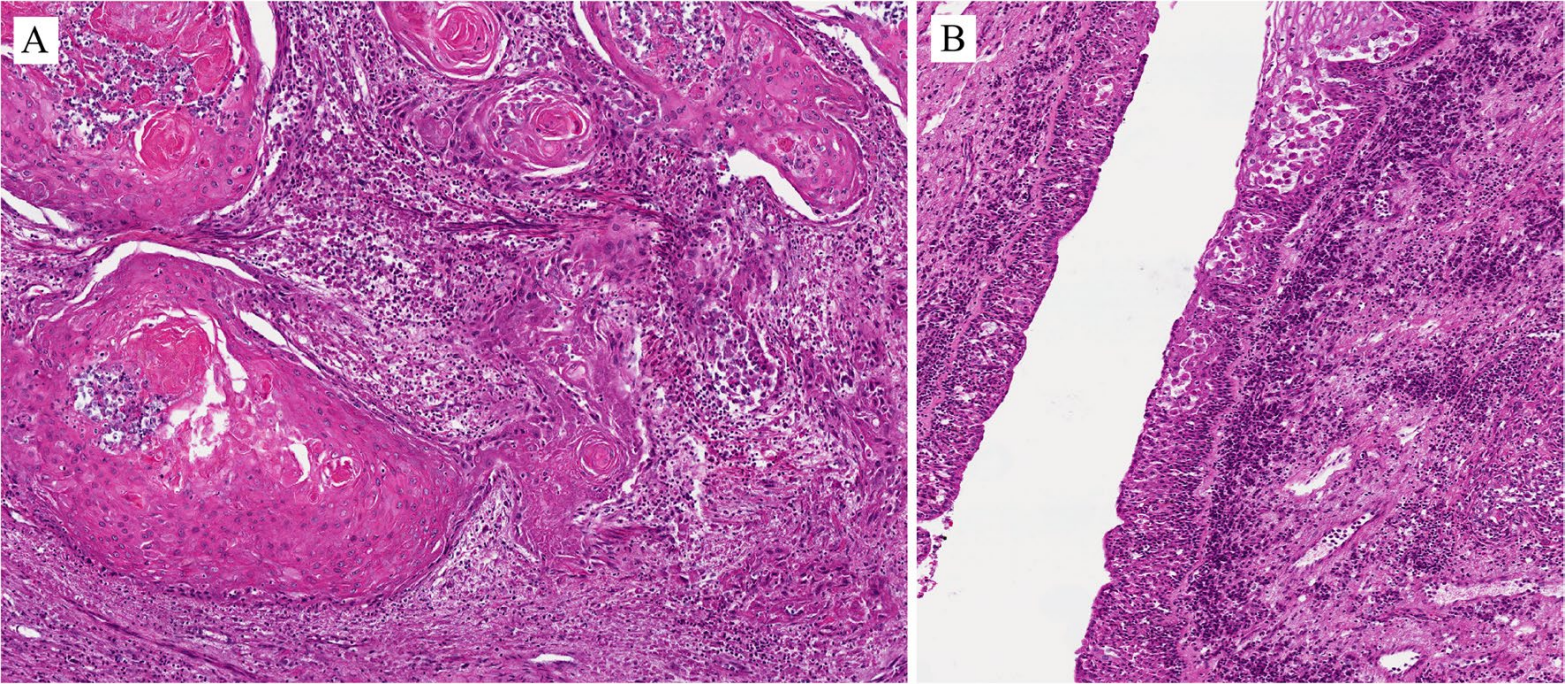
Metachronous well differentiated squamous cells carcinoma of the female urethra

SCC of the urethra can be associated with high-risk HPV infection both in female and male patients. In particular, HPV16 or HPV18 may be detected in up to 60% of SCCs in women, whereas in men about 30% of SCCs are positive for HPV16 [8, 9]. The presence of HPV does not mean that the tumor is aggressive. In fact, some HPV16 positive tumors may have a more favorable prognosis. Not all SCCs are HPV related. For instance, tumors in the bulbar urethra are usually negative. Figures 8–10 are examples of SCC involving the urethra.

Figure 8 is an example of SCC originating in the female urethra; its location was the distal one-third. The tumor invades the periurethral muscle (Fig. 8A). Its morphology is shown in the image: SCC with very few keratin pearls, i.e., the tumor is poorly differentiated (Fig. 8B). By immunohistochemistry, the lesion is p16 positive (Fig. 8C). HPV18, a high-risk HPV, was detected with molecular techniques.

Figure 9 is another example of SCC. This was in the male urethra. The tumor shows an in-situ component (Fig. 9A) and invasion of the corpus spongiosum (Fig. 9B). Both the non-invasive and invasive components are composed of cells with basaloid features (Fig. 9C). In the invasive component, there is an abrupt transition from vital cells to necrosis (Fig. 9D). This is seen in the basaloid variant of SCC. Molecular techniques have shown the presence of HPV16.

Figure 10A is an example of a well differentiated SCC of the distal part of the female urethra associated, distally, with an in situ component with pagetoid features (Fig. 10B). The patient had SCC of the vulva in her past clinical history. The tumor can be considered a direct extension from the vulva (metachronous), rather than primary in the urethra.

### Adenocarcinoma

The adenocarcinoma of the urethra can be subdivided into two groups, conventional or nonclear cell adenocarcinoma and clear cell adenocarcinoma. Conventional adenocarcinoma derives from the surface epithelial lining. It shows a male predominance with a ratio M:F of 2–3 to 1. Clear cell adenocarcinoma is considered of Müllerian origin, i.e., thought to arise from preexisting Müllerian precursors within the urinary bladder and urethra, and shows a female predominance with a ratio of 1 to 4.6 [10, 11].

#### Conventional adenocarcinoma

**Fig. 11:**
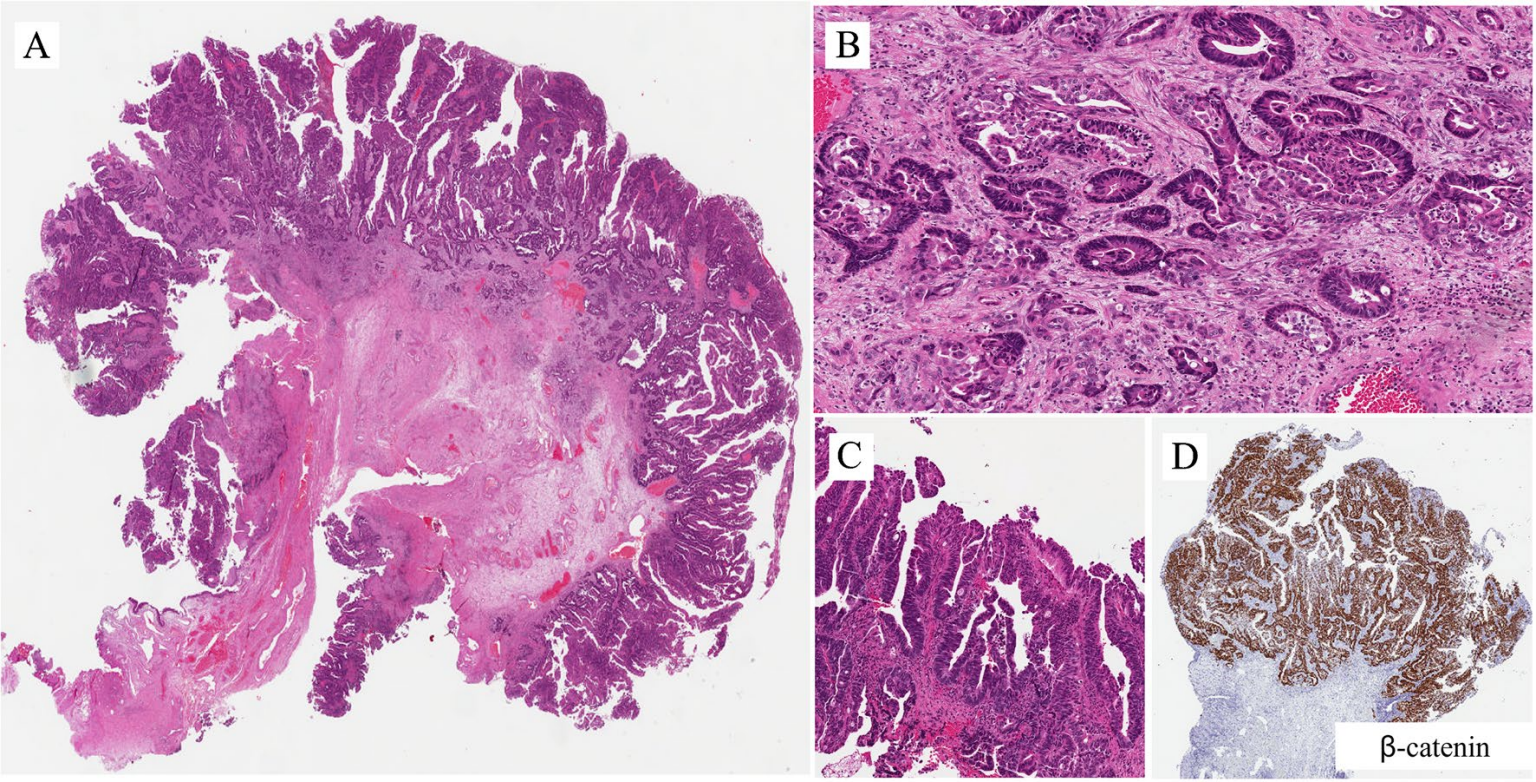
Adenocarcinoma of enteric type in the male urethra

**Fig. 12:**
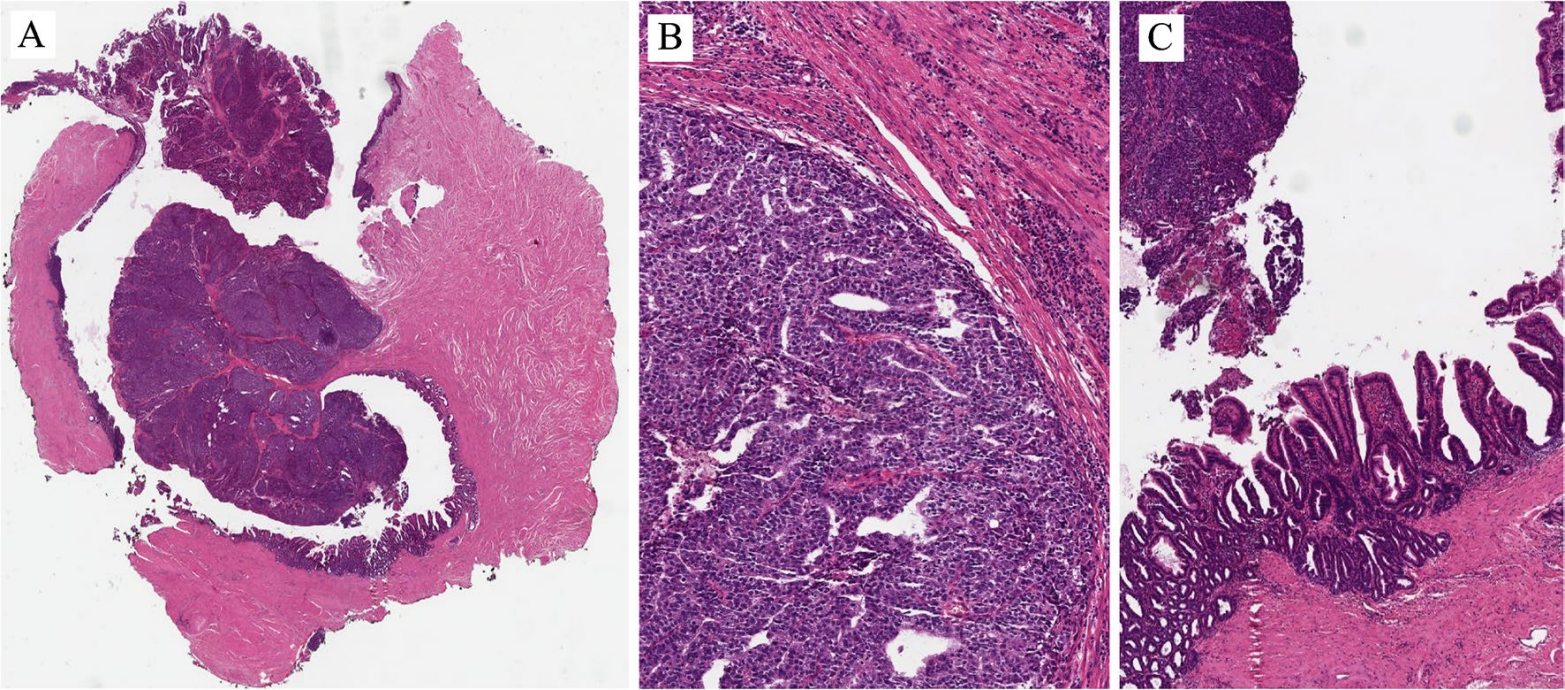
Adenocarcinoma in a diverticulum in the female urethra

**Fig. 13:**
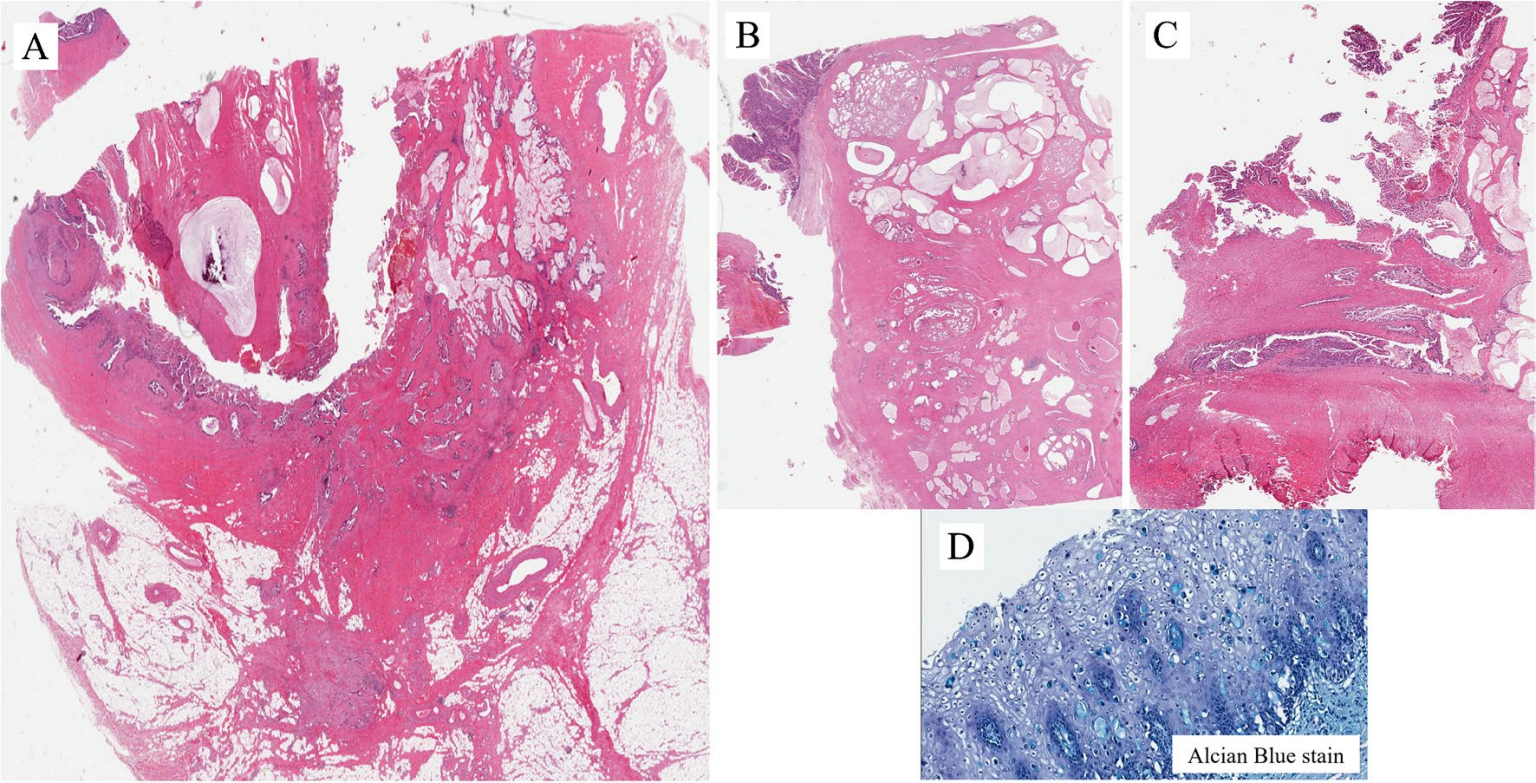
Synchronous Adenocarcinoma of the prostatic and penile urethra

**Fig. 14:**
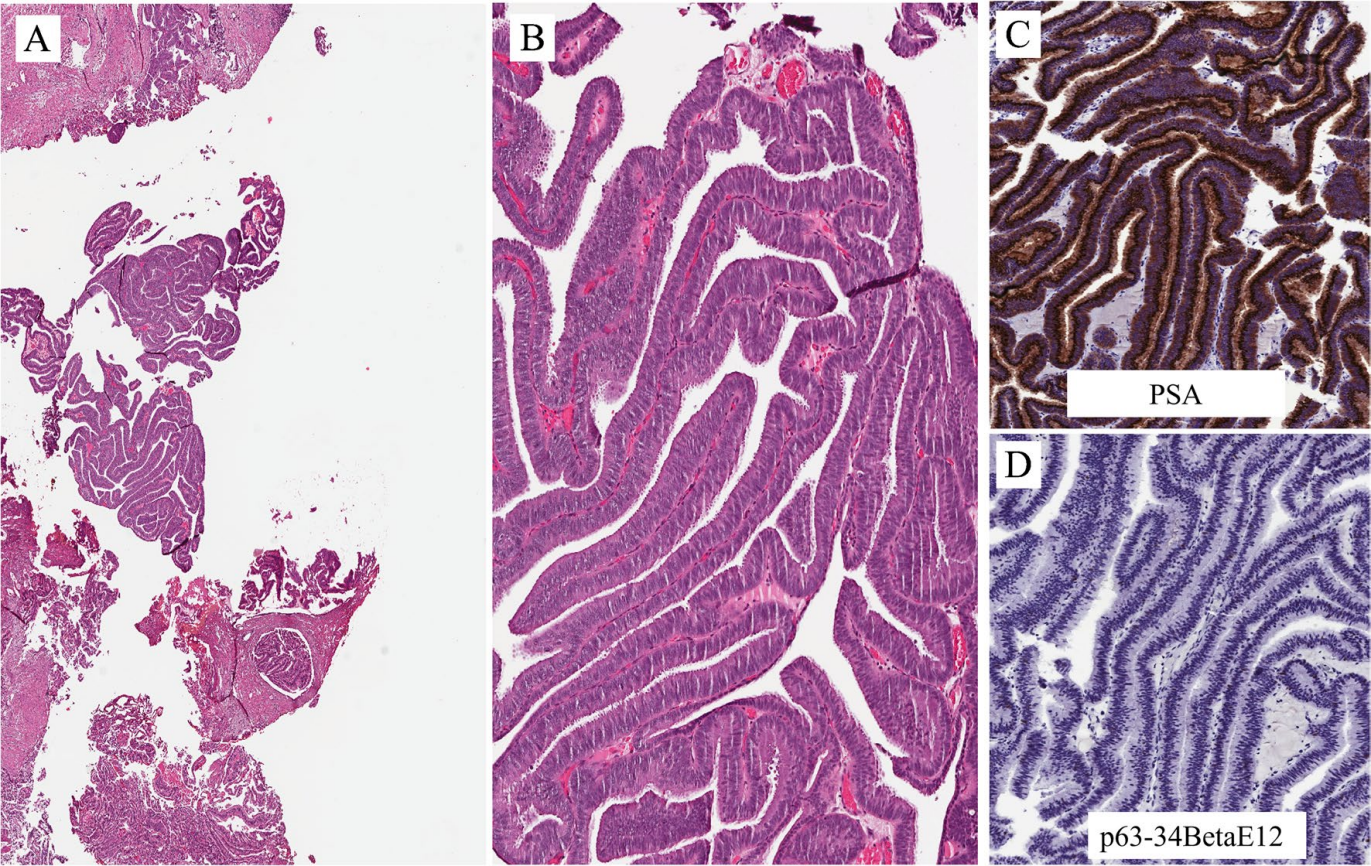
Ductal prostate adenocarcinoma with extension through periurethral ducts into the urethra

Conventional adenocarcinoma of the urethra shows a range of patterns similar to that originating in the bladder. The most common is the enteric (colonic) type adenocarcinoma, and adenocarcinoma, not otherwise specified. There are also rarer histologic subtypes such as signet ring cell, mucinous (or colloid), hepatoid, and mixed forms. ADCs are usually graded following criteria similar to ADCs in other organs (i.e., degree of differentiation). Rare cases of ADC originating in the accessory glands can appear as conventional ADC. Rare tumors have a resemblance to prostate cancer. Some types of adenocarcinomas (such as the enteric type) are associated with (or arise from) intestinal metaplasia, including villous adenoma. Such lesions arise from the surface epithelium and are seen in association with longstanding inflammatory insult, diverticulum or stricture [12, 13]. Figures 11–14 are examples of conventional adenocarcinoma of the urethra.

Figure 11A is an example of adenocarcinoma of enteric type in the male urethra. It is similar to that usually seen in the colon (Fig. 11B). It invades the subepithelial connective tissue. Grading is that used in the adenocarcinoma of the colon. The mucosa adjacent to the invasive neoplasms shows intestinal metaplasia and high-grade dysplasia (Fig. 11C); very similar lesions are seen in the bladder and even in the colon in association with adenocarcinoma. Immunohistochemistry was applied in order to confirm the primary nature of the adenocarcinoma. The tumor was intensely stained for beta catenin (Fig. 11D), at the level of the cell membrane. Such information is of fundamental importance for confirming the primary nature. A study published by Liang Cheng and his group [14] aimed at distinguishing primary adenocarcinoma of the urinary bladder from secondary involvement by colorectal adenocarcinoma. The study reported that Beta catenin is membranous in 92% and nuclear in the remaining 8% of primary bladder adenocarcinomas, whereas is nuclear in 92% of cases of secondary involvement of the bladder by colorectal cancer. However, there are many exceptions to this nuclear vs. membranous pattern distribution in colorectal cancer.

Figure 12A is an example of primary adenocarcinoma originating in a diverticulum in a female urethra. According to the literature, ADC is the most common histologic type of primary urethral carcinoma arising in a setting of diverticulum. It is a poorly differentiated adenocarcinoma (Fig. 12B) with membranous expression of beta catenin. The mucosa adjacent to the ADC shows intestinal metaplasia with dysplasia (Fig. 12C). A paper, published by Dr Hale and collaborators, described such an association, that is, ADC and intestinal type metaplasia [15].

Figure 13 is another case of adenocarcinoma of enteric type and with mucinous features in a patient with a history of bladder adenocarcinoma. The neoplasm is present in the bladder where it invades the perivesical fat (Fig. 13A) and involves the prostatic urethra (Fig. 13B) and the penile urethra (Fig. 13C). This can be considered a direct extension of the tumor to the urethra from the urinary bladder. It is synchronous. The surgical procedure included total penectomy. Histological samples of the glans show, as you can see in the alcian blue stained slide, atypical cells with cytoplasmic vacuoles present in the thickness of the squamous epithelium (Fig. 13D). This particular case was reported in the literature by Dr Cimadamore as extramammary Paget disease of the penis closely mimicking the penile analog of stratified mucin-producing intraepithelial lesion [16].

The histological images in Fig. 14A are from a TUR of a velvety lesion on the surface of the posterior urethra in proximity to the verumontanum. Histologically, the lesion shows a villous appearance with a minor degree of atypicality, reminiscent of villous adenoma with dysplasia (Fig. 14B). The lesion was on the surface of the mucosa with some extension within periurethral ducts. The differential diagnosis includes villous adenoma with dysplasia, prostatic-type polyp, and ductal prostate adenocarcinoma with extension through periurethral ducts into the urethra. Immunohistochemistry has a great role in refining the diagnosis. PSA is positive (Fig. 14C). This excludes villous adenoma. It remains prostatic-type polyp vs. ductal ADC. Immunohistochemistry for p63+34betaE12 shows that basal cells are not present (Fig. 14D). This excludes the diagnosis of prostatic-type polyp. The final diagnosis is ductal ADC, a lesion that has to be considered in the differential diagnosis of conventional adenocarcinoma of the urethra and its associated lesions or precursors.

#### Clear cell adenocarcinoma

CCA is characterized by pattern heterogeneity within the same neoplasm: solid, tubular, tubulocystic, or papillary patterns. Cytologically, it shows abundant clear to eosinophilic cytoplasm that contains glycogen and little or no mucin. Occasionally, the luminal location of the nuclei imparts a hobnail appearance to the cells. The nuclei in clear cell adenocarcinoma vary in morphology from high grade to low grade. The former is more frequently observed. When low grade is present, the neoplasm resembles nephrogenic adenoma and the differential diagnosis may be challenging in a small biopsy. However, there are morphologic criteria that can help us differentiate NA from CCA [17, 18]. Hartmann et al. reported a case with molecular evidence for progression of NA of the urinary bladder to CCA [19]. The exact relationship of CCA to NA is still controversial.

**Fig. 15:**
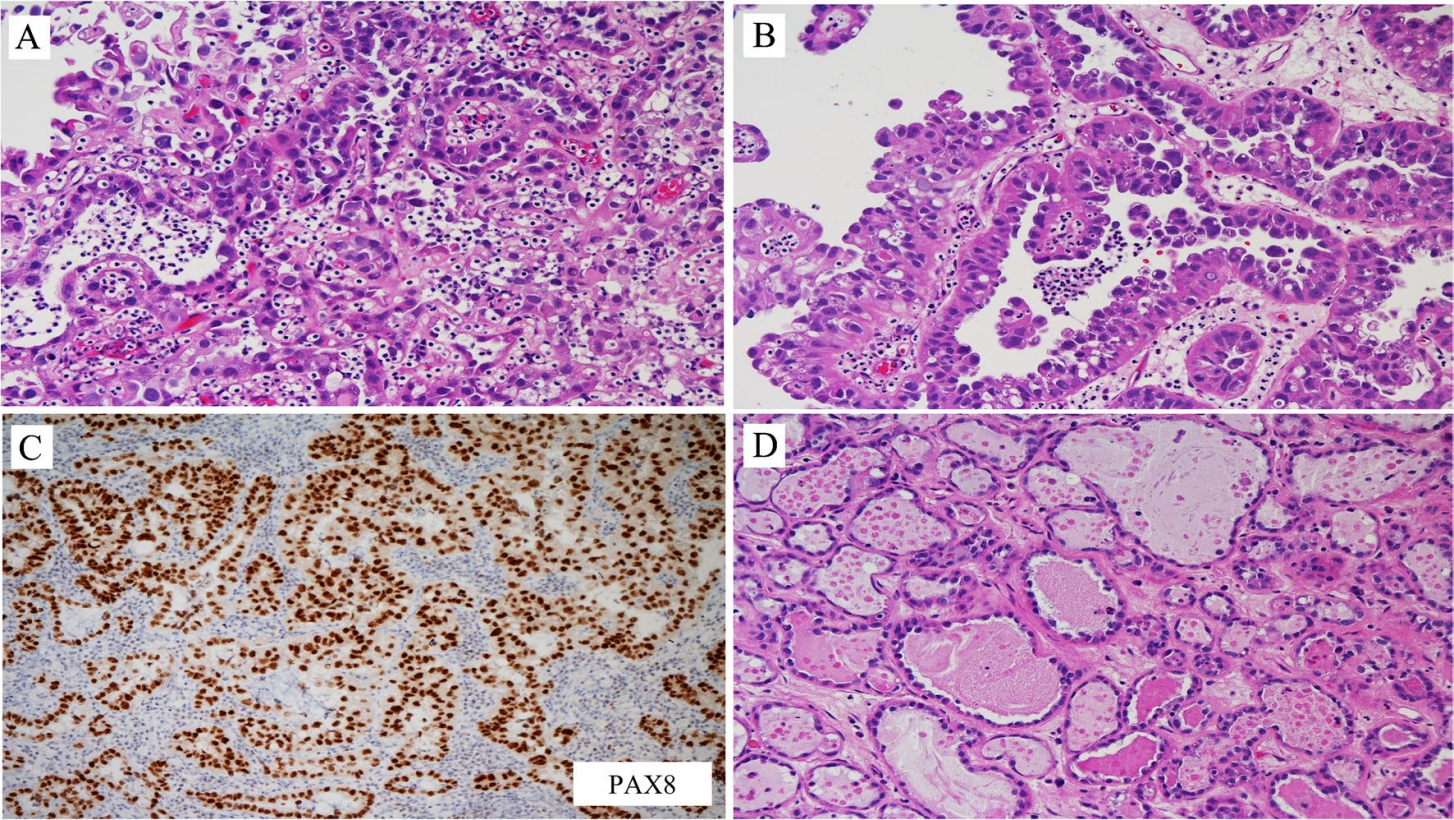
Clear cell adenocarcinoma of the urethra

**Fig. 16:**
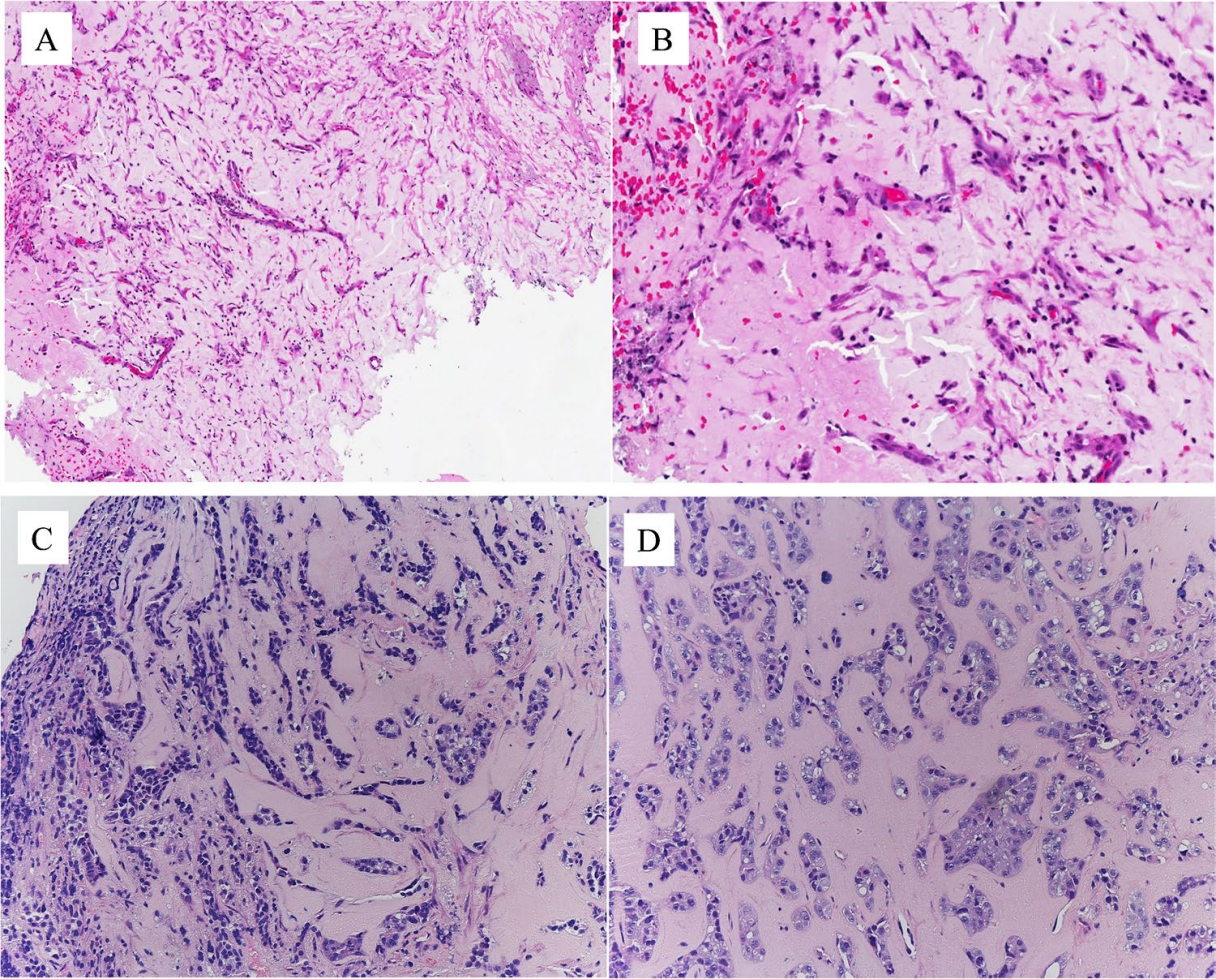
Fibromyxoid nephrogenic adenoma and hyalinizing clear cell carcinoma

**Fig. 17:**
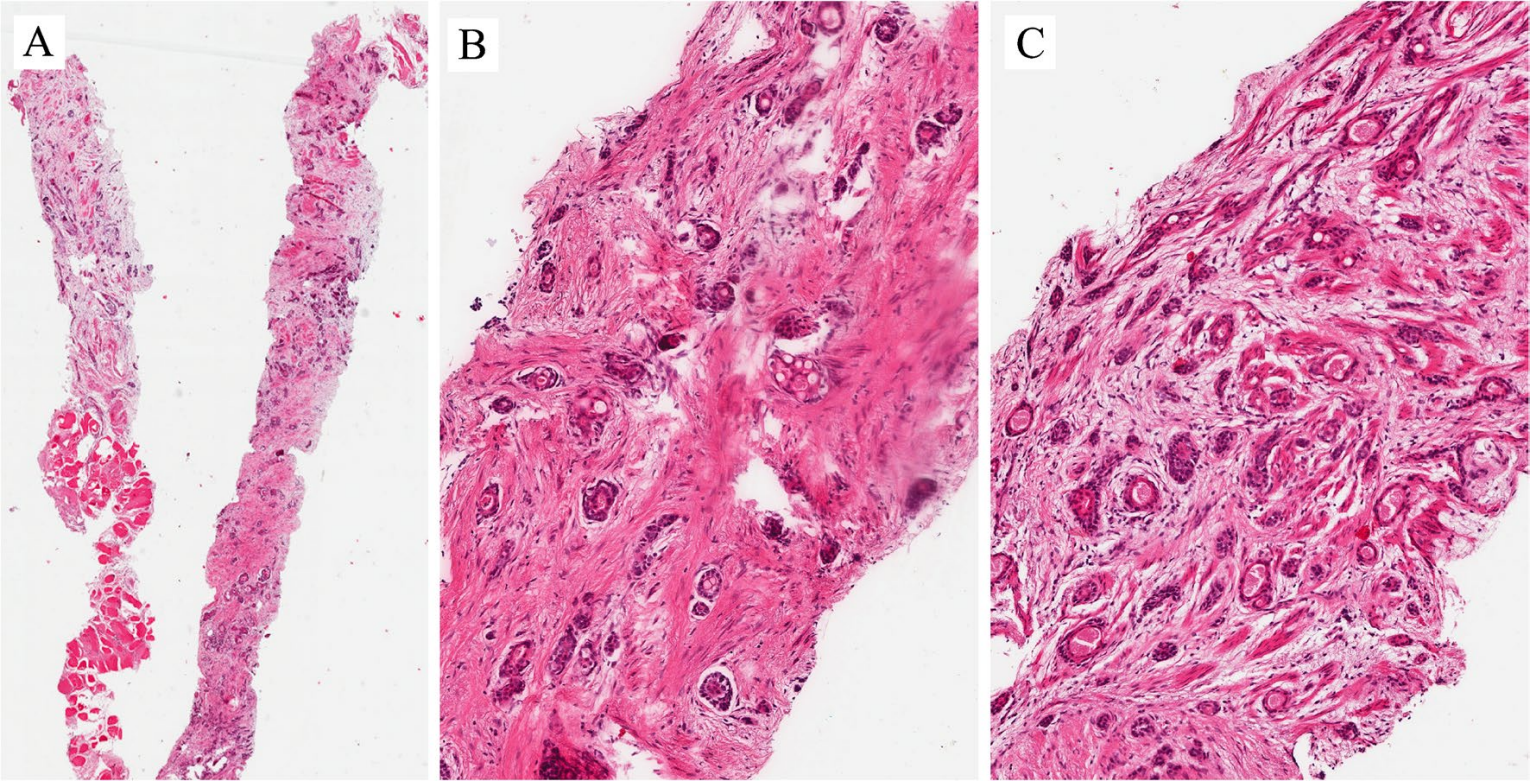
Cowper gland adenocarcinoma

Figures 15–17 are examples of CCA of the urethra. These images of Fig. 15 are from a typical case of clear cell adenocarcinoma, both from the architectural and cellular point of view (Fig. 15A). In some areas, a hobnail appearance due to the luminal location of the nuclei is seen (Fig. 15B). PAX8 is positive in this type of adenocarcinoma (Fig. 15C), whereas it is negative in conventional adenocarcinoma. The image in Fig. 15D is from the same case shown in Fig. 15A. It represents an area with nephrogenic adenoma-like clear cell adenocarcinoma. A final diagnosis is feasible based on the morphologic evaluation of all the patterns present in the neoplasia. In cases of clear cell adenocarcinoma of the bladder and urethra diffusely mimicking nephrogenic adenoma, as suggested in a paper by Dr Epstein and colleagues, the key features discriminating between nephrogenic adenoma-like clear cell adenocarcinoma and nephrogenic adenoma include occasional clear cells, more prominent pleomorphism especially hyperchromatic enlarged nuclei, and extensive muscular invasion [17].

The differential diagnosis becomes more complicated with unusual variants of NA and of CCA. Figure 16A and B are from a case of fibromyxoid nephrogenic adenoma, a rare variant of NA that consists of compressed spindle-shaped renal epithelial cells in a fibromyxoid background. In nearly a quarter of the cases, fibromyxoid nephrogenic adenoma extends beyond the lamina propria. It can be pure in morphology or intermixed with typical NA or with malignant neoplasms. There is a very rare case of variant of clear cell carcinoma or adenocarcinoma, occurring in different organs, including the urethra, and that can mimic fibromyxoid nephrogenic adenoma (Fig. 16C and D). The architecture and cell type are similar to classic clear cell adenocarcinoma, including PAX8 positivity. It consists of clear cells arranged in nests or trabeculae with a hyalinized stroma. The degree of cytological atypia is much higher than in NA. This case was seen in the bulbous urethra. The patient developed metastasis with the same features. It is called *hyalinizing clear cell carcinoma.*

In rare occasions, CCA can mimic metastatic clear cell renal cell carcinoma, mainly for the presence of cells with clear cytoplasm, including PAX8 expression. However, the architecture and marker expression are different between the two types of carcinomas. CAIX is positive in the clear cell renal cell carcinoma and is negative in CCA.

## Tumors originating in the accessory glands

Adenocarcinoma originating in the accessory glands, in particular, in the Cowper and Littré glands in men and Skene glands in women is exceedingly rare. The best indicators of accessory gland origin are absence of neoplastic changes in the urethral epithelium and partial involvement of recognizable periurethral glands. Very limited information about biological behavior: prognosis is related to stage.

### The Cowper gland adenocarcinoma

The Cowper gland adenocarcinoma occurs in the proximal (bulbomembranous) urethra, and the Littré gland adenocarcinoma originates in the periurethral glands of the penile urethra. These two types of adenocarcinomas can be distinguished on the basis of their location and also morphology [20].

Cowper gland adenocarcinoma usually shows the morphology of adenoid cystic carcinoma, similar to that seen in the salivary glands. This type of tumor is characterized by the chromosomal translocation typically found in the salivary tumor, i.e., MYB-NFIB gene fusion. Mucin either intracytoplasmic or as frank mucinous component, as in adenocarcinoma with enteric features, is rare. Immunohistochemistry shows that the neoplastic cells are CK7 positive and PSA negative [21].

Figure 17A–C show an example of the Cowper gland adenocarcinoma. At low magnification, the neoplasm invades the striated sphincter (external urethral sphincter) (Fig. 17A). The other images show the neoplasms at a higher magnification, and an adenoid cystic pattern is shown, associated with a fibrous stromal reaction, apart from hypoxic areas (Figure 17B and C).

### Littré gland adenocarcinoma

The Littré gland adenocarcinoma shows papillary or tubulopapillary architecture. The cells are columnar with abundant cytoplasm and mucinous vacuoles. Nuclei are large and oval, without marked pleomorphism. Rare cases may show morphologic features similar to those originating from the surface, in particular ADC with an enteric pattern. Immunohistochemistry does not help in making a final diagnosis of this type of accessory gland adenocarcinoma. The neoplastic cells are positive for CK7 and negative for PSA [22].

**Fig. 18:**
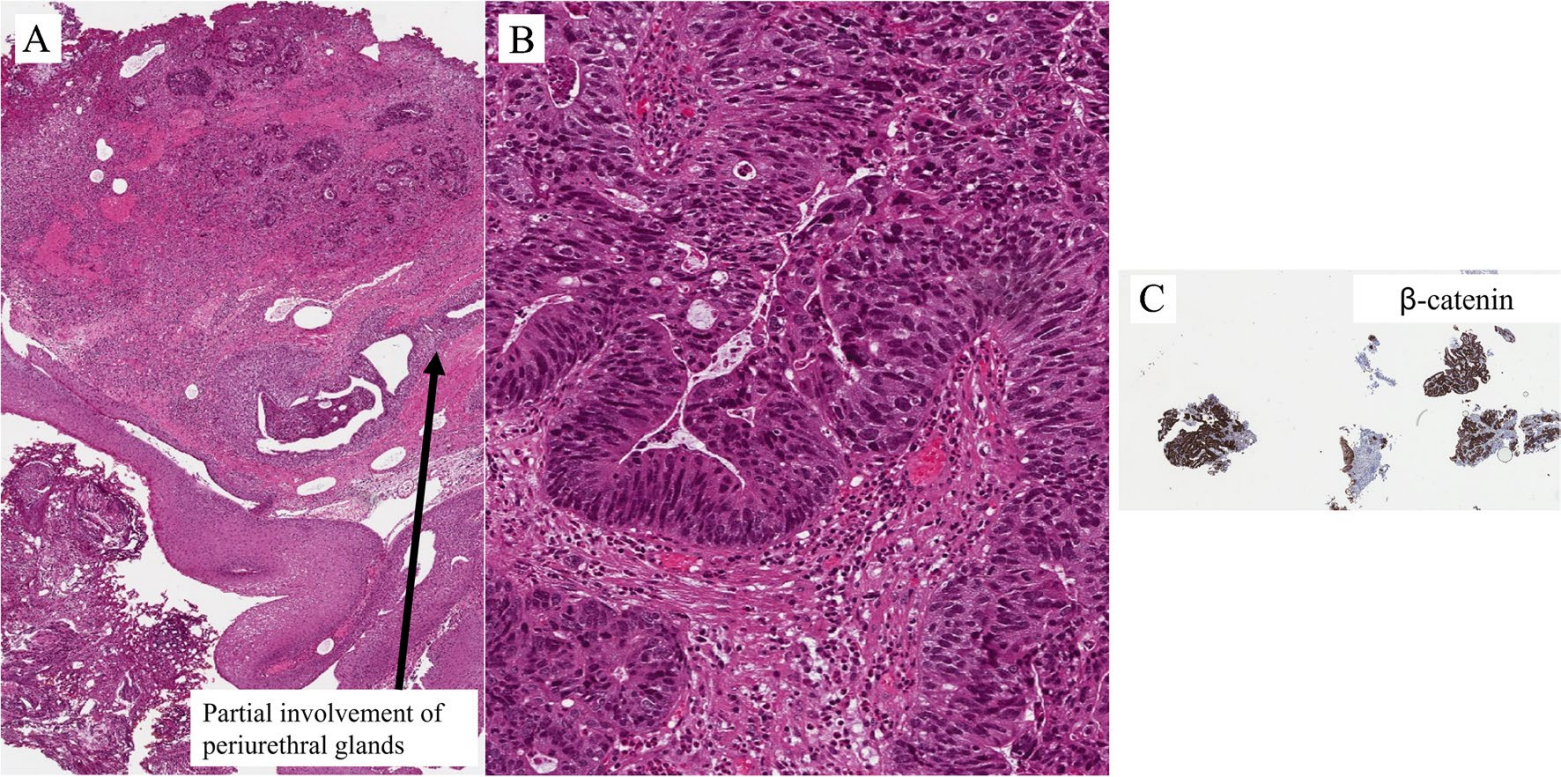
Littré gland adenocarcinoma

Figure 18A shows images from a biopsy of a neoplasm in the penile urethra. The surface is lined by a squamous epithelium. The subepithelial connective tissue shows an adenocarcinoma with mucous vacuoles (Fig. 18B). Immunohistochemistry shows membranous expression of beta catenin (Fig. 18C) as seen in the primary adenocarcinoma of the urethra. In the subepithelial connective tissue, adjacent to the neoplasia, there are some glandular structures partly lined by a squamous epithelium and, more distantly from the surface, a glandular epithelium. At a higher magnification, the glandular epithelium shows some degree of atypicality. This might represent partial involvement of recognizable periurethral glands.

### Skene gland adenocarcinoma

The Skene gland adenocarcinoma in the female urethra shows histologic similarities with prostatic acinar adenocarcinoma. Various patterns can be seen, such as papillary, tubulopapillary, tubular, solid, and cribriform patterns. The neoplasia is positive immunohistochemically for prostate markers, such as PSA, PSAP, NKX3.1, AMACR, and P501S. Mutation and loss of heterozygosity of the *PTEN* gene can be detected [23].

**Fig. 19:**
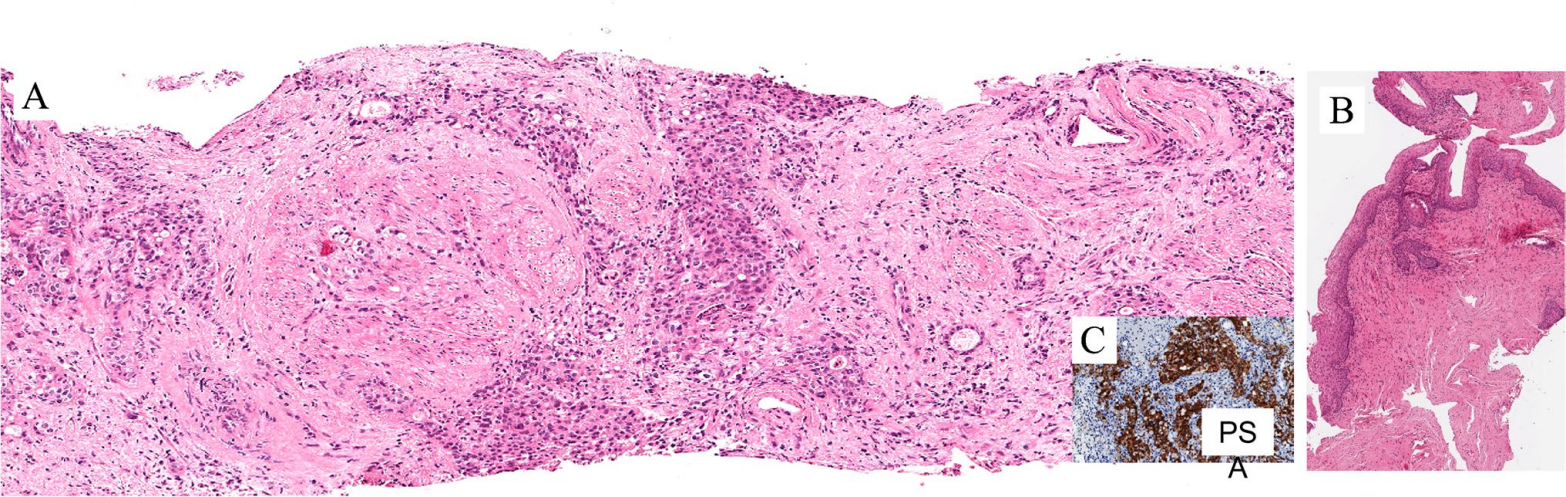
Skene gland adenocarcinoma

These images of Fig. 19A are from a case of Skene gland adenocarcinoma invading the subepithelial connective tissue and the periurethral muscle (T2). The surface epithelium is not affected by the neoplasm (Fig. 19B). Immunohistochemistry shows a diffuse and intense staining for PSA (Fig. 19C). When the neoplasm is located distally, close to the external sphincter, a Bartholin gland carcinoma should be considered in the differential diagnosis. Bartholin gland carcinoma is negative for PSA. This helps in making a final diagnosis. Serum PSA is elevated in women with Skene gland adenocarcinoma. Changes in the serum level are a reliable marker to monitor response to therapy.

## Conclusions

An accurate diagnosis, grading and staging are essential for determining adequate treatment strategies and outcome. An accurate diagnosis can be based on the latest revision of the WHO blue book. Staging is one of the most important prognostic factors (together with tumor location). It is based on the TNM classification of tumors of the urethra (included in the Cancer Staging Manual, Eight Edition, 2017) [24] (Tables 2 and 3). Tumor location is also important in terms of prognosis. Five-year survival in women is 51% for proximal tumors vs. 6% for distal tumors. In men, it is 50% for proximal tumors vs. 20% for distal tumors [25].

**Table 2:** Staging of the tumors of the prostatic urethra (pT Category)

Tis: Carcinoma in situ involving the prostatic urethra or periurethral or prostatic ducts without stromal invasion	
T1: Tumor invades urethral subepithelial connective tissue immediately underlying the urothelium	
T2: Tumor invades the prostatic stroma surrounding ducts either by direct extension from the urothelial surface or by invasion from prostatic ducts	
T3: Tumor invades the periprostatic fat	
T4: Tumor invades other adjacent organs (e.g., extraprostatic invasion of the bladder wall, rectal wall)

**Table 3:** Staging of the tumors of the male penile urethra and female urethra (pT Category)

TX: Primary tumor cannot be assessed	
T0: No evidence of primary tumor	
Ta: Non-invasive papillary carcinoma	
Tis: Carcinoma in situ	
T1: Tumor invades subepithelial connective tissue	
T2: Tumor invades any of the following: corpus spongiosum, periurethral muscle	
T4: Tumor invades adjacent organs (e.g., invasion of the bladder)	

Specimen handling and reporting can be based on the CAP (College of American Pathologists) protocol for the examination of the tumors of the urethra and the reporting guide according to the ICCR (International Collaboration on Cancer Reporting) [26]. The EAU guidelines on primary urethral carcinoma made recommendations for morphologic diagnosis, grading and staging [27]. The recommendation was strong for the 2017 TNM staging and for the most recent WHO blue book revision. In particular, the guidelines suggested that specimen handling should follow the general rules published by the International Collaboration on Cancer Reporting (Also known as ICCR) [28].
